# Reproduction in the Maxillary Bones

**Published:** 1853-10

**Authors:** 


*Reproduction in the Maxillary Bones.*—The Southern Journal of Medical and Physical Sciences, for September, 1853, contains a very interesting article on "Repair" and reproduction in the maxillary bones, by the editor of the dental department of that publication. It embodies, besides some very sensible physiological remarks of the author, the opinions of numerous writers upon the subject; some of which are adverse to the doctrine that the loss of the whole, or any part of either of these bones, is ever replaced with true osseous structure, while others, founded upon well authenticated facts, establish most conclusively, that the reproductive energies of the economy are, under certain circumstances, exercised here as well as in other parts of the osseous system. The author quotes, in a note to his article, an editorial from the October No., 1852, of this Journal, in which the doctrine of reproduction in the maxillary bones is opposed, and attributes the authorship of it to the senior editor. In connection with the opinion here expressed, he refers to our remarks on the loss of the alveoli in our Principles and Practice of Dental Surgery, and putting the two together, he arrives at the conclusion, that we believe, "the maxillary bones are incapable of regeneration, either in whole or in part."

The editorial quoted by Dr. Wood, from this Journal, was written by the junior editor, and only expressed his own individual views, as derived from the general tenor of the authorities which he had, at that time, read upon the subject. The senior editor has long been of the opinion, that the power of regeneration is, under certain circumstances and conditions of the surrounding structures, exercised in the maxillary as well as in other bones of the body. Although an example of the kind has never fallen under his own immediate observation, several well authenticated cases are on record. Dr. E. S. Bennett, of Charleston, S. C., communicated to him, through Dr. J. A. Cleveland, of the same place, the history of a case which was published in the first number of the fifth volume of this Journal. The subject was a negro child, about two years and a half old, and the loss occasioned by necrosis, extended from the right inferior canine tooth to the articulation on the left side. The necrosed bone was removed by Dr. Bennett, and is now in the Museum of the Baltimore College of Dental Surgery.

"This case," says Dr. B. "settles the question definitely, as to whether nature, of herself, is capable of reproducing the bony structures entirely; in this case, not only the whole bone has been reproduced, but dentition also—being now armed with two formidable grinders."

A case of reproduction of the body of the inferior maxillary bone, extending from one coronoid process to the other, was described to the senior editor several years ago, by the late Dr. Snyder, of Baltimore. The subject was a boy, about twelve years of age. The loss of the primitive bone was occasioned by mercurial salivation. But in this case, there was no reproduction of teeth.

The history of a case of a somewhat similar character, communicated by Wm. Jones, M. D., of Kenton, Ohio, was published in the last number of this Journal.

But, while we are convinced that nature sometimes makes a successful effort to replace the loss of a part, and occasionally, even of the whole of the inferior maxillary, examples of such reproductive energy here, it must be confessed, are exceedingly rare, and there is not, we believe, a single well authenticated case on record, to show that the loss of alveoli simply, whether occasioned by mechanical violence, necrosis, or any other cause, are ever replaced.

